# New species of *Paracolpenteron* n. gen. and *Ancyrocephalus* (Monogenea, Dactylogyridae) inhabiting the urinary bladder and gills of the Maya needlefish *Strongylura hubbsi* (Beloniformes, Belonidae) from Chiapas, Mexico

**DOI:** 10.1051/parasite/2018053

**Published:** 2018-11-16

**Authors:** Edgar F. Mendoza-Franco, Juan M. Caspeta-Mandujano, Carlos Ramírez-Martínez

**Affiliations:** 1 Instituto de Ecología Pesquerías y Oceanografía del Golfo de México (EPOMEX), Universidad Autónoma de Campeche Av. Héroe de Nacozari No. 480 CP 24029 San Francisco de Campeche Campeche México; 2 Facultad de Ciencias Biológicas y Centro de Investigaciones Biológicas, Laboratorio de Parasitología de Animales Silvestres, Universidad Autónoma del Estado de Morelos, Avenida Universidad No. 1001 Colonia Chamilpa 62209 Cuernavaca Morelos México; 3 Laboratorio de Producción Acuícola, Facultad de Medicina Veterinaria y Zootecnia, Universidad Autónoma de Nuevo León (UANL) Ex-Hacienda el Canadá CP. 66050 México

**Keywords:** *Ancyrocephalus*, *Paracolpenteron* n. gen., n. sp., freshwater fish, biogeography

## Abstract

Parasitological examination of the maya needlefish *Strongylura hubbsi* Collette (Belonidae) from the Rio Lacantún basin in the Montes Azules Biosphere Reserve, Chiapas, Mexico showed that specimens were parasitized by two monogenean species in two different sites: *Paracolpenteron hubbsii* n. gen., n. sp in the urinary bladder and *Ancyrocephalus chiapanensis* n. sp in the gill lamellae. *Paracolpenteron hubbsii* differs from other dactylogyrid species without a haptoral anchor/bar complex infecting the urinary systems, gills and nasal cavities by the general morphology of hooks, a dextral vaginal opening, a tubular male copulatory organ comprising a base from which a coiled shaft arises in counterclockwise direction, and an unarticulated Y-shaped accessory piece. *Ancyrocephalus chiapanensis* n. sp. resembles *Ancyrocephalus cornutus* William & Rogers, 1972 from the gills of *Strongylura marina* from Florida from which it differs in possessing a twisted tube of the male copulatory organ (curved in *A. cornutus*), ventral bar with cavities on the ends (cavities absent in *A. cornutus*) and by the size of the ventral (length 31–34 μm vs. 24–27 μm in *A. cornutus*) and dorsal (length 25–28 μm vs. 18–22 μm in *A. cornutus*) anchors. These new monogeneans are described herein and their biogeography is briefly discussed based on the previous phylogenetic hypotheses concerning the host family.

## Abstract (fr)

L’examen parasitologique du poisson *Strongylura hubbsi* Collette (Belonidae) du bassin du Rio Lacantún dans la réserve de biosphère de Montes Azules, Chiapas, Mexique, a révélé qu’il était parasité par deux espèces de monogènes dans deux sites différents : *Paracolpenteron hubbsii* n. gen., n. sp dans la vessie et *Ancyrocephalus chiapanensis* n. sp sur les lamelles branchiales. *Paracolpenteron hubbsii* diffère des autres espèces de Dactylogyridae sans complexe ancre / barre haptorale qui infectent les systèmes urinaires, les branchies et les cavités nasales par la morphologie générale des crochets, une ouverture vaginale dextre, un organe copulateur mâle tubulaire comprenant une base à partir de laquelle se forme une tige enroulée dans le sens antihoraire et une pièce accessoire non articulée en forme de Y. *Ancyrocephalus chiapanensis* ressemble à *Ancyrocephalus cornutus* n. sp. William & Rogers, 1972 des branchies de *Strongylura marina* de Floride, dont il diffère par la possession d’un tube torsadé de l’organe copulateur mâle (incurvé chez *A. cornutus*), une barre ventrale avec des cavités aux extrémités (cavités absentes chez *A. cornutus*) et par la taille des ancres ventrale (longueur 31–34 μm vs. 24–27 chez *A. cornutus*) et dorsale (longueur 25–28 μm vs. 18–22 chez *A. cornutus*). Ces nouveaux monogènes sont décrits ici et leur biogéographie est brièvement discutée sur la base d’hypothèses phylogénétiques antérieures concernant la famille hôte.

## Introduction

In Mexico, monogeneans from sites other than the gills and skin of fishes have rarely been studied, with a few dactylogyrid species described and/or recorded, i.e., *Enterogyrus malmbergi* Bilong Bilong, 1988 in the stomach of the introduced tilapia *Oreochromis niloticus* (L.) and the native cichlid *Cichlasoma callolepis* (Regan) (now *Thorichthys callolepis*) from Santa Anita Lagoon in the state of Tabasco [13]; *Pavanelliella scaphiocotylus* Kritsky & Mendoza-Franco, 2003 in the nasal cavity of the catfish *Rhamdia guatemalensis* (Günther) (Heptapteridae) from a cenote (= sinkhole) in the Yucatán Peninsula [19]; *Pseudempleurosoma carangis* Yamaguti, 1965 and *Pseudempleurosoma gibsoni* Santos, Mourão & Cárdenas, 2001 in the rectum of the puffer fish *Sphoeroides testudineus* (Linnaeus) (Tetraodontidae) and the pyloric ceca of the cobia *Rachycentron canadum* (Linnaeus) (Rachycentridae), respectively, from the northern coast of the Yucatan Peninsula [27]; and *Cacatuocotyle chajuli* Mendoza-Franco, Caspeta-Mandujano & Salgado-Maldonado, 2013 in the external surface of the anal opening of the characid *Astyanax aeneus* (Günther) from the Rio Lacantún basin in the state of Chiapas [25] (see [28]). During studies carried out between February and August 2015 on the parasites of fishes from the Rio Lacantún basin, two undescribed dactylogyrid species were found at two sites in the Maya needlefish *Strongylura hubbsi* Collette (Belonidae): *Paracolpenteron hubbsii* n. gen., n. sp. in the urinary bladder and *Ancyrocephalus chiapanensis* n. sp. in the gill lamellae. In the present paper, both new species are described.

## Materials and methods

Following approval from the Ethics Committee of the Autonomous University of Nuevo Leon (UANL), and after obtaining a permit from the Secretaría del Medio Ambiente y Recursos Naturales (SEMARNAT), Mexico (permit numbers: FAUT-017 and SGPA/DGVS/03492), specimens of *S. hubbsi* were captured by hook-and-line and throw nets between February and August 2015 in the Rio Lacantún basin in the state of Chiapas (16°09′96.6″N, 90°95′56.8″W). Live fish were sacrificed by puncturing the brain region (a needle is introduced dorsally via the eye socket and moved about to destroy the brain and spinal cord) [29]. The gills of each fish were removed and placed in finger bowls containing 4–5% formalin solution to fix any of the ectoparasites that might be present. The internal cavity of each fish was exposed by an incision made along the middle of abdomen from anus to mouth. The monogenean specimens were removed from the urinary bladder and preserved in 4% formalin. Subsequently, parasites preserved in formalin were isolated and stained with Gomori’s trichrome and mounted in Canada balsam. In addition, some specimens were mounted in a mixture of lactic-acid (LA) and glycerine-ammonium picrate (GAP), and then remounted in Canada balsam [25] to obtain measurements and line drawings of haptoral structures and the copulatory complex. All other measurements were obtained from unflattened specimens stained with Gomori’s trichrome stain. Drawings were made with the aid of a drawing tube using a DM2500 Leica microscope with Nomarski interference contrast. Measurements, all in micrometers, represent straight-line distances between extreme points and are expressed as the mean followed by the range and number (*n*) of structures measured in parentheses; body length includes that of the haptor. The direction of the coil (clockwise vs. counterclockwise) of the copulatory organ was determined using the procedure suggested by Kritsky et al. [16]. Type specimens are deposited in the National Helminthological Collection of Mexico (CNHE).

## Results

Subclass Polyonchoinea Bychowsky, 1937.

Order Dactylogyridea Bychowsky, 1937.

Dactylogyridae Bychowsky, 1933.

### *Paracolpenteron* n. gen. (Figs. 1–5)

urn:lsid:zoobank.org:act:C0825287-DF80-4E3C-B22B-D75248C2605E

**Figures 1–5.**
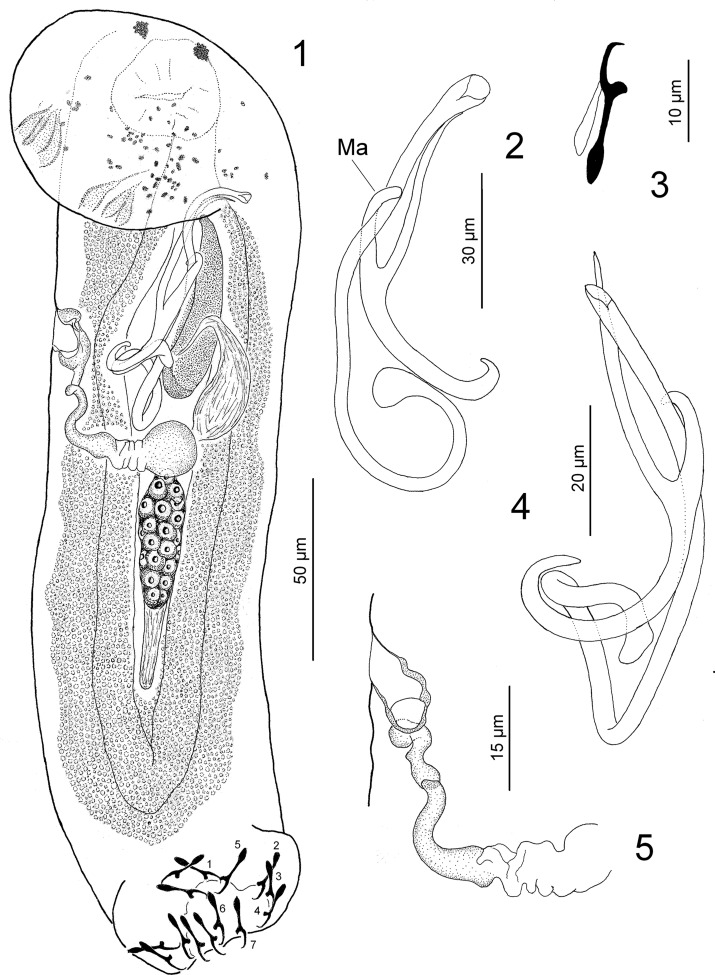
*Paracolpenteron hubbsii* n. gen, n. sp. from the urinary bladder of *Strongylura hubbsi*. 1: whole-mount (composite, ventral view); 2: copulatory complex (dorsal view) (Ma, medial arm); 3: hook; 4: copulatory complex (ventral view); 5: vagina. Figures are drawn to the following scales: 50 μm (Fig. 1), 30 μm (Fig. 2), 10 μm (Fig. 3), 20 μm (Fig. 4), and 15 μm (Fig. 5).

Type-species: *Paracolpenteron hubbsii* n. sp.

Etymology: The genus name refers to the resemblance of the new taxon to *Acolpenteron* Fischthal & Allison, 1941, whose members parasitize the urinary systems of cypriniform and perciform fishes from the southern Neotropical realm and the Nearctic and Palearctic regions of the Northern Hemisphere.

Diagnosis: Body comprising body proper (cephalic region, trunk, and peduncle) and haptor. Tegument smooth. Cephalic region rounded, lacking lobes. Head organs and cephalic glands present. Eyes present. Mouth sub-terminal, midventral, prepharyngeal; pharynx a muscular glandular bulb; esophagus, intestinal ceca 2, confluent posterior to gonads, lacking diverticula. Common genital pore midventral, posterior to intestinal bifurcation. Gonads intercecal, overlapping; testis dorsal to germarium. Proximal vas deferens not observed; seminal vesicle a simple dilation of distal vas deferens; prostatic reservoir present. Copulatory complex comprises unarticulated male copulatory organ (MCO) and accessory piece. MCO tubular, sclerotized, comprising base from which arises coiled shaft in counterclockwise direction. Seminal receptacle pregermarial; vaginal aperture dextroventral. Vitellaria in trunk, peduncle, absent in regions of other reproductive organs. Haptor cup-shaped, armed with seven pairs of similar hooks, each with shank comprising two subunits; proximal subunit expanded. Anchors, bars, 4A’s absent. Type- and only species: *Paracolpenteron hubbsii* n. gen., n. sp. from the urinary bladder of *Strongylura hubbsi* (Belonidae).

### *Paracolpenteron hubbsii* n. sp.

urn:lsid:zoobank.org:act:41E05DC5-900F-4458-B410-ABEDED3D45B1

Type-host: Maya needlefish *Strongylura hubbsi* Collette 1974 (Beloniformes: Belonidae)

Site of infection: Urinary bladder.

Type locality and collection date: Rio Lacantún basin in the state of Chiapas, Mexico (19°09′96.6″N, 90°95′56.8″W), February 2015.

Specimens deposited: holotype, 16 paratypes in CNHE (10728 and 10729, respectively).

Etymology: The specific epithet is derived from the specific name of the type host.

#### Description (Figs. 1–5)

Diagnosis (based on eight specimens fixed in a mixture of LA and GAP and nine specimens stained with Gomori’s trichrome): Body 294 (220–343; *n* = 8) long; greatest width 71 (65–78; *n* = 9) near midlength or in anterior trunk. Three pairs of bilateral head organs; cephalic glands indistinct. One to four eyespots; accessory chromatic granules that are similar in size and shape are scattered in the cephalic region. Pharynx spherical, 27 (23–30; *n* = 9) in diameter; esophagus moderately long. Testis, 37 (30–40; *n* = 5) long, 16 (8–32; *n* = 4) wide. Seminal vesicle pyriform, lying ventral to the prostatic reservoir. Prostatic reservoir elongate pyriform, extending toward the left side of the copulatory complex. MCO a coiled tube with a poorly defined counterclockwise ring, 81 (62–90; *n* = 11) long; base with rounded margin at opening. Accessory piece 64 (55–72; *n* = 7) long, Y-shaped, proximally recurved, lying along distal portion of MCO with medial arm (see Ma in Fig. 2) wrapping around MCO. Germarium 29 (20–54; *n* = 4) long, 17 (11–21; *n* = 6) wide; oviduct, ootype, uterus not observed. Vaginal aperture marginal, at level of the midportion of the copulatory complex; vaginal vestibule large, sclerotised, lying diagonally on right side of body; vaginal duct sclerotized, arising from proximal end of vaginal vestibule from which it descends forming a loop followed by highly compressed section before extending to spherical seminal receptacle. Vitellaria dense, extending posteriorly from posterior margin of pharynx in lateral fields of trunk, into median field of peduncle, to level of anterior margin of haptor. Haptor, 64 (55–75; *n* = 8) wide. Four hooks (pairs 1 and 5) ventral and more centrally located in haptor; hooks pairs 2–4 on bilateral haptoral pads, directed ventrally along lateral margin of haptor; remaining hooks (pairs 6 and 7) situated slightly more dorsal in haptor than pairs 2, 3, 4 (see haptor in Fig. 1). Hook 19 (18–20; *n* = 14) long, with protruding thumb, delicate point; filamentous hook loop (FH loop) 80% shank length.

#### Differential diagnosis

After comparing and examining original descriptions of dactylogyrid genera whose species do not possess a haptoral anchor/bars complex [i.e., species of *Acolpenteron* Fischthal & Allison, 1941 (syn. *Pseudacolpenteron* Bychowsky & Gussev, 1955) (see [20]); *Anacanthorus* (Mizelle & Price 1965) Kritsky et al. 1992; *Anacanthoroides* Kritsky & Thatcher 1976; *Anonchohaptor* (Mueller 1938) Kritsky, Leiby & Shelton, 1972; *Icelanonchohaptor* Leiby, Kritsky & Peterson, 1972; *Kritskyia* (Kohn 1990) Boeger et al. [2]; *Octouncuhaptor* Mendoza-Franco, Roche & Torchin, 2008; *Pavanelliella* (Kritsky & Boeger, 1988) Kritsky & Mendoza-Franco, 2003; and *Telethecium* Kritsky, Every & Boeger, 1996, all from characiform, perciform, clupeiform, cypriniform and siluriform fishes], with that of the dactylogyrid found in the urinary bladder of *S. hubbsi* (Beloniformes), we concluded that it is a new dactylogyrid that does not fit into any genera listed above (see [2, 17–19, 21–23, 26, 30, 32]). Therefore, *Paracolpenteron* n. gen. is proposed to accommodate the new species.

Additionally, *P. hubbsii* n. gen., n. sp. differs from other dactylogyrids (see genera listed above) in having a dextroventral vaginal aperture (lacking vagina in *Anacanthorus*; vagina sinistral in *Anacanthoroides*, *Kritskyia*, *Pavanelliella* and *Telethecium*); overlapping gonads (tandem in *Anacanthorus* and slightly overlapping in *Octouncuhaptor*); an unarticulated MCO to accessory piece (articulated in *Octouncuhaptor* and *Telethecium*); and by lacking a uterus with well-developed metraderm and “larval hooks” 4A’s (present in species of *Anacanthorus*; as hooks “reduced” in *Anacanthoroides*); pronounced cephalic lappets and germarium loops around the right limb of the intestine (present in *Anonchohaptor*); and a hook-like MCO and V or U-shaped sclerite associated with hooks (present in *Anonchohaptor* and *Icelanonchohaptor*) (see [1, 2, 4, 6, 11, 14, 18, 19, 23, 26]).

*P. hubbsii* n. gen., n. sp. most resembles species of *Acolpenteron* infecting the urinary systems of freshwater cypriniform and perciform fishes, in having overlapping gonads, a copulatory complex comprising an unarticulated Y-shaped accessory piece with the MCO base, a dextroventral vaginal pore and haptor cup-like with 14 similar hooks (see [7–9]). *P. hubbsii* n. gen., n. sp. differs from species of this latter genus in having a comparatively small body (220–343 long vs. 383–998 and greatest width 65–78 vs. 90–202) and shortened between the testis and the posterior confluence of the intestinal ceca [extended in *A. ureteroecetes* Fischthal & Allison 1941 (type species of the genus) and *A. australe* Viozzi & Brugni, 2003]; smaller hooks (18–20 long vs. 23–32); a coiled MCO with a poorly defined counterclockwise ring vs. a straight tubular, twisted or arcing tube in *A. catostomi* Fischthal & Allison 1942, *A. nephriticum* Gvozdev 1945, *A. willifordensis* Fayton & Kritsky 2013, and *A. ureteroecetes*; one prostatic reservoir vs. two prostatic reservoirs in *A. australe*, *A. catostomi*, *A. willifordensis*, and *A. ureteroecetes*; and by the position of the vaginal opening (i.e., at level of the midportion of the copulatory complex vs. right margin posterior to MCO in *A. willifordensis* and *A. ureteroecetes* [8, 12, 33, 36].

### *Ancyrocephalus chiapanensis* n. sp.

urn:lsid:zoobank.org:act:3E1116B8-BDAF-403A-B7D8-88D77F55216B

Type host: Maya needlefish *Strongylura hubbsi* Collette 1974 (Beloniformes: Belonidae)

Type locality and collection date: Rio Lacantún basin in the state of Chiapas, Mexico (19°09′96.6″N, 90°95′56.8″W), February 2015.

Site of infection: Gill lamellae.

Specimens deposited: holotype, 20 paratypes in CNHE (10730 and 10731, respectively).

Etymology: The specific name of this species relates to the state of Chiapas.

#### Description (Figs. 6–12)

**Figures 6–12.**
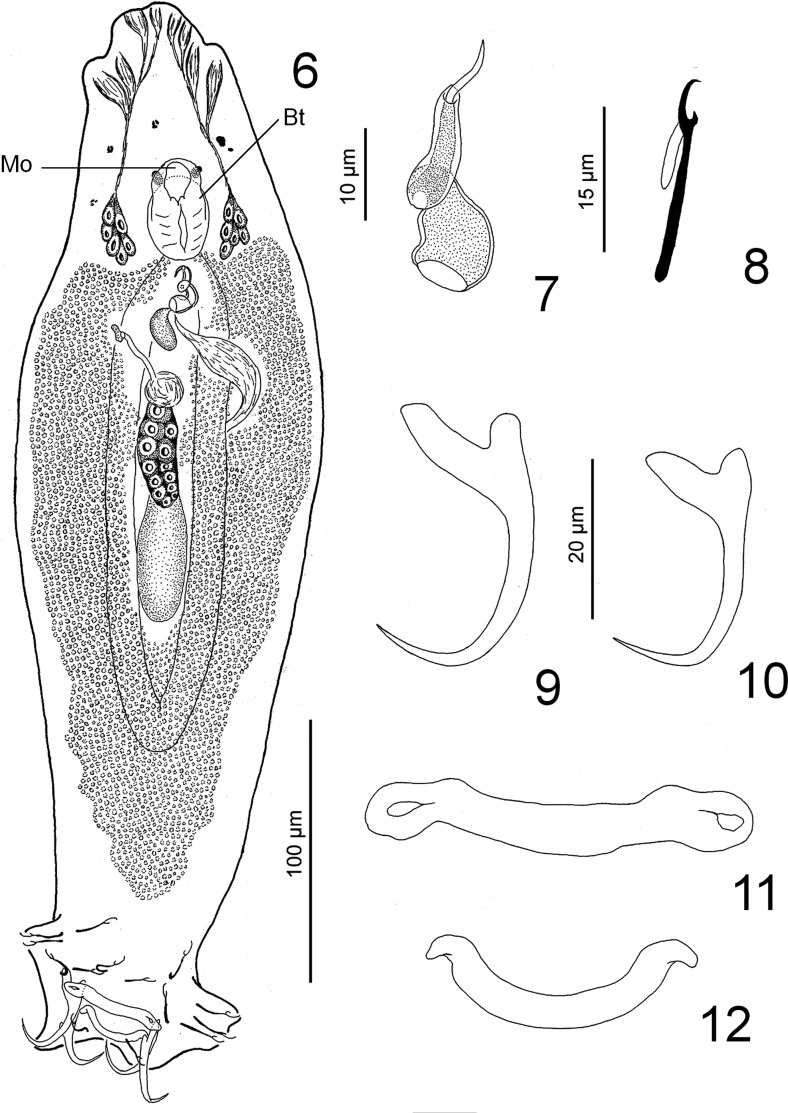
*Ancyrocephalus chiapanensis* n. sp. from the gills of *Strongylura hubbsi*. 6: whole-mount (composite, ventral view); 7: copulatory complex; 8: hook; 9: ventral anchor; 10: dorsal anchor; 11: ventral bar; 12: dorsal bar. Figures are drawn to the following scales: 100 μm (Fig. 6), 10 μm (Fig. 7), 15 μm (Fig. 8) and 20 μm (Figs. 9–12).

Diagnosis (based on four specimens fixed in a mixture of LA and GAP and 17 specimens stained with Gomori’s trichrome). Body fusiform, 347 (290–430; *n* = 19), greatest width 105 (90–120; *n* = 16) at midlength. Tegument smooth. Cephalic lobes poorly developed, two bilateral pairs of head organs; cephalic glands lateral and posterolateral to pharynx. Eyespots two (infrequently four). Mouth midventral, at level of anterior margin of pharynx (see Mo in Fig. 6), opening into buccal tube (see Bt in Fig. 6) which is slightly extending posteriorly along body midline to pharynx. Pharynx ovate, 25 (21–30; *n* = 17) wide; esophagus absent; intestinal ceca 2, confluent posterior to testis, lacking diverticula. Gonads slightly overlapping, testis overlaps posterior margin of germarium dorsally, 56 (32–93; *n* = 11) long, 20 (15–24; *n* = 10) wide. Vas deferens looping left intestinal cecum; seminal vesicle a simple dilation of vas deferens. Prostatic reservoir pyriform. MCO 21 (20–25; *n* = 16) long, with bulbous or bell-shaped base from which arises a shaft in counterclockwise direction before forming a twisted tube; termination of the MCO appears as a tapered tube within a tube, this latter tube (accessory piece) serving as guide for MCO. Germarium fusiform, 33 (27–40; *n* = 4) long, 20 (17–22; *n* = 3) wide; oviduct, ootype and uterus not observed. Genital pore midventral. Vaginal aperture dextral, submarginal; vaginal canal a delicate tube, poorly sclerotized; spherical seminal receptacle located on anterior margin of germarium. Vitellaria dense, coextensive with ceca. Peduncle broad, tapered posteriorly; haptor subhexagonal, 87 (76–95; *n* = 10) wide with well-developed lateral lobes. Anchors similar; each with well-developed superficial root, short rounded deep root, elongate shaft, gently arcing, elongate point extending just past level of tip of superficial root; ventral anchor 32 (31–34; *n* = 16) long, base 16 (15–17; *n* = 10) wide; dorsal anchor 27 (25–28; *n* = 8) long, base 11 (11–12; *n* = 7) wide. Ventral bar 42 (36–48; *n* = 11) long, rod-shaped, slightly arcuate, with cavities on ends. Dorsal bar 32 (30–35; *n* = 6) long, U-shaped, with tapered ends posteriorly directed. Hooks dissimilar in size; each with upright thumb, curved shaft and evenly curved point and shank composed of one subunit (expanded gradually toward proximal end); FH loop about 40% shank length; hook pair 5–15 (*n* = 3) long; hook pairs 1–4, 6 and 7–20 (*n* = 7) long.

#### Differential diagnosis

*Ancyrocephalus chiapanensis* n. sp. resembles *Ancyrocephalus cornutus* William & Rogers, 1972 on *Strongylura marina* (Walbaum) from Florida, USA. *A. cornutus* is a species that has remained in *Ancyrocephalus* (s.l.) because no clear synapomorphies have been identified to place it in a genus. Similarly, *A. chiapanensis* n. sp. lacks such identifying features. Therefore, the generic assignment of *A. chiapanensis* n. sp. and *A. cornutus* is provisional and based on agreement with the diagnosis provided by Williams [34]. *A. cornutus* and *A. chiapanensis* n. sp., both on their respective host species of *Strongylura,* share the following characteristics: anchors with a well-developed superficial root, short rounded deep root, elongate shaft and point extending just past level of tip of superficial root; MCO inside of a tube acting as an accessory piece (nested tube) (as a club-shaped accessory piece in *A. cornutus*). Although not originally described in *A. cornutus*, the copulatory complex depicted in Figure 7 from the original description of this species suggests a bulbous-shaped base of the MCO, similar to that of *A. chiapanensis* n. sp. (see [35]). While convergence cannot be discounted, these above-mentioned morphological characters indicate that the two monogenean species are morphologically very similar, suggesting that they may have coevolved alongside their respective host species. *A. chiapanensis* n. sp. differs from *A. cornutus* in having a twisted MCO and accessory piece (curved in *A. cornutus*); two intestinal ceca confluent posterior to gonads (ceca not confluent in *A. cornutus*); a ventral bar with cavities on the ends (absent in *A. cornutus*); and by the size of the ventral (length 31–34 vs. 24–27 in *A. cornutus*) and dorsal (length 25–28 vs. 18–22 in *A. cornutus*) anchors (see [27], present study). While a strict definition of *Ancyrocephalus* (s.l.) remains wanting, erection of a new genus to accommodate *A. chiapanensis* n. sp. and *A. cornutus* will additionally depend on examination of type specimens of *A. cornutus* (not currently available) or on newly collected specimens from *S. marina* in Florida. This latter will allow us to clarify whether the ceca are confluent or not as well as the position of other organs, i.e., vagina which was not described in the original description of *A. cornutus*.

In terms of other features, *A. chiapanensis* n. sp. exhibits some similarity with *Xenentocleidus xenentodoni* (Jain) Tripathi, Agrawal & Pandey 2007 from *Xenentodon cancila* (Hamilton) and *Hemirhamphiculus exserocephalus* Kritsky 2018 from *Tylosurus gavialoides* (Castelnau), both belonid hosts from India and Australia, respectively (see [15, 31]). These three monogenean species possess a dextroventral and submarginal vaginal aperture (marginal in *H. exserocephalus*) and the shaft of the MCO in a counterclockwise direction. *A. chiapanensis* n. sp. differs from *X. xenentodoni* in having slightly overlapping gonads (tandem in *X. xenentodoni*); a twisted MCO (coiled tube in *X. xenentodoni*); an unarticulated accessory piece and MCO (accessory piece articulated with the base of the MCO in *X. xenentodoni*); by lacking anchor roots connected by a threadlike sclerotized piece (present in *X. xenentodoni*); hooks with a shank composed of two subunits (present in *X. xenentodoni*); and tegument transversally striated ventrally at the region of the copulatory complex (present in *X. xenentodoni*) (see [15, 30]).

## Discussion

The Río Lacantún basin of southeastern Mexico and northern Guatemala in the Neotropical Region is characterized by numerous derivatives of marine species adapted to freshwater [10]. The evolutionary radiation of fish in this region indicates old ichthyofauna with a high degree of endemism at the generic and suprageneric levels [3]. An example of this in the area is the endemic freshwater needlefish *S. hubbsi* and their monogeneans. Most needlefishes are marine, but 12 species are restricted to freshwater and several species of *Strongylura* move long distances into freshwater. The freshwater fish species mentioned above include species in three genera (*Belonion* Collette, *Potamorrhapis* Gunther and *Pseudotylosurus* Fernández-Yépez, with seven endemic species from South American rivers) plus two species of *Strongylura* in freshwaters of Central and South America, and one genus (*Xenentodon*), with two species plus *Strongylura krefftii* (Günther) in South-east Asian freshwaters [5, 24]. As stated in Lovejoy & Collette [24], based on morphological and molecular tools, freshwater needlefishes from the Atlantic basins do not make up a monophyletic group and *Strongylura* is clearly polyphyletic. Instead, the genus consists of several small monophyletic species groups, such as *S. marina* (Atlantic) and *S. exilis* (Girard) (Pacific) as a sister taxon of *S. hubbsi*. In accordance with this, the present study shows that *A. chiapanensis* n. sp. infecting *S. hubbsi* (Pacific) exhibits some morphological similarity (see Differential diagnosis section for *A. chiapanensis* n. sp.) with a species assigned to *Ancyrocephalus* (s.l.), *A. cornutus*, from *S. marina.* This morphological evidence supports Lovejoy & Collette′s clade containing *S. marina* within a sister taxon of *S. hubbsi* and suggests that these monogeneans from *S. hubbsi* and *S. marina* form a monophyletic group from which they could have coevolved with their hosts. This latter hypothesis is consistent with the sister taxon of *S. hubbsi* that consists of an Atlantic and Pacific species pair (*S. marina* and *S. exilis*, respectively) from which *S. hubbsi* is thought to have diverged prior to the last connection between these oceans (3 Mya) [5], when freshwater ancestors of needlefishes were originally distributed from the Atlantic drainage of the Rio Usumacinta of Mexico and Guatemala to the Pacific slopes of South America, and the Atlantic drainages of South America [24]. However, a phylogenetic hypothesis for members of all dactylogyrids infecting fishes in the New World (i.e., needlefishes) is necessary to formally address the hypothesis mentioned above.
